# Understanding the interplay between mild traumatic brain injury and cognitive fatigue: models and treatments

**DOI:** 10.2217/cnc-2017-0003

**Published:** 2017-10-27

**Authors:** Glenn R Wylie, Laura A Flashman

**Affiliations:** 1 Kessler Foundation, Rocco Ortenzio Neuroimaging Center, 1199 Pleasant Valley Way, West Orange, NJ 07052, USA; 2 Department of Physical Medicine & Rehabilitation, New Jersey Medical School, Rutgers University, Newark, NJ 07101, USA; 3 The Department of Veterans’ Affairs, The War Related Illness & Injury Center, New Jersey Healthcare System, East Orange Campus, East Orange, NJ 07018, USA; 4 Dartmouth Hitchcock Medical Center, Dartmouth College, Geisel School of Medicine, Lebanon, NH 03756, USA

**Keywords:** cognitive fatigue, mild TBI, REVIEW

## Abstract

Nearly 2 million traumatic brain injuries occur annually, most of which are mild (mTBI). One debilitating sequela of mTBI is cognitive fatigue: fatigue following cognitive work. Cognitive fatigue has proven difficult to quantify and study, but this is changing, allowing models to be proposed and tested. Here, we review evidence for four models of cognitive fatigue, and relate them to specific treatments following mTBI. The evidence supports two models: cognitive fatigue results from the increased work/effort required for the brain to process information after trauma-induced damage; and cognitive fatigue results from sleep disturbances. While there are no evidence-based treatments for fatigue after mTBI, some pharmacological and nonpharmacological treatments show promise for treating this debilitating problem. Future work may target the role of genetics, neuroinflammation and the microbiome and their role in complex cognitive responses such as fatigue.

With some 1.8 million traumatic brain injuries (TBIs) occurring annually in the USA [1], and a growing number occurring in countries such as India [2], the incidence of TBI is alarmingly high. The majority of these TBIs are classified as closed head injuries, in which there is blunt trauma and/or rapid acceleration/deceleration of the brain, resulting in multifocal lesions and diffuse brain damage, the outcome of which is a variety of cognitive and physical deficits [3]. Moreover, the overwhelming majority of all TBIs are mild in severity (mTBI). Although it was once believed that there were essentially no long-term consequences following mTBI, it is now known that mTBI can induce several long-term sequelae including headaches, difficulty in concentrating and fatigue [4].

Immediately following an mTBI, during the acute period (i.e., less than 3 months after the injury), the most frequently reported complaints are poor memory, sleep disturbances and fatigue [5]. Headaches are also common (reported in 71.5% of individuals who have sustained a mTBI [6]), but fatigue is the most severe symptom [6]. Estimates of the incidence of fatigue after TBI vary from 21 to 73%, depending on the characteristics of the studied population (e.g., severity of injury, time since injury, sampling of patients, etc.) and the method used to identify fatigue (e.g., single item or fatigue scales) [7–13]. While little work has been done specifically evaluating the prevalence of cognitive fatigue in the mTBI population, this is beginning to change. For example, Norrie *et al*. investigating the prevalence of fatigue across time in mTBI, found the prevalence of fatigue to be initially 68% (1 week post-mTBI) and to decrease to 38% over the next 3 months, with severity correlating with prevalence [14]; no distinction was made between types of fatigue. However, even 6 months after mTBI, 34% of individuals still reported significant levels of fatigue [14]. This accords well with other work reporting that 6 months after the injury, 32% of individuals who have sustained a mTBI report severe fatigue (relative to only 12% of people with minor injuries not involving brain trauma) and fatigue degrades their quality of life [15]. Moreover, while the type of injury (complicated vs uncomplicated mTBI [a complicated mTBI involves either a depressed skull fracture and/or an intracranial abnormality related to the trauma while an uncomplicated mTBI does not [16]]) does not predict the amount of fatigue individuals subsequently experience [17], whether or not individuals experience fatigue does predict the duration of postconcussive symptoms [18,19]. Thus, fatigue following mTBI appears to be both prevalent and persistent, and has real implications for the quality of life of individuals who sustain mTBI.

The aim of this paper is to review current models of cognitive fatigue after mTBI, as well as current treatments. In the following review, we will begin with an overview of the problem of fatigue in mTBI, then we will move into a description of the major models that have been proposed to account for fatigue in mTBI, and we will conclude with a section on potential treatments for fatigue in mTBI, with available evidence. A systematic review was performed to identify studies of mTBI and fatigue using PubMed from its inception through 16 May 2016 using MeSH terms and keywords. No language, type of study or date restriction was applied in the initial search. All citations were examined and nonrelevant studies were excluded based on their titles and abstracts. Studies were additionally excluded if they were animal studies or case reports. Manuscripts of all potentially included studies were obtained, and those with a focus on TBI and dimensions of fatigue, models of fatigue, or treatment were read and incorporated into this review as appropriate. See Figure 1 for visual presentation of the search strategy and total manuscripts included.

**Figure 1.**
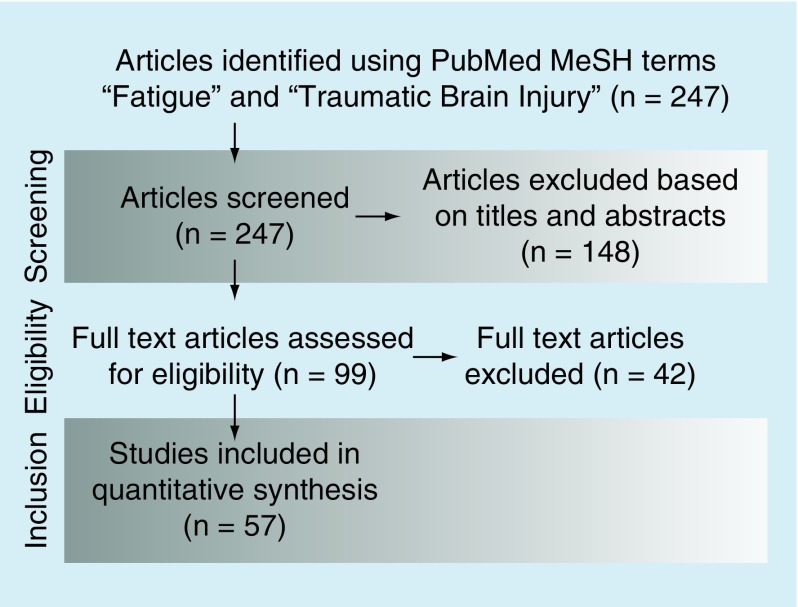
**A flow diagram of the search methodology used in this review.**

In the last century, researchers have had great difficulty in both defining and assessing fatigue [20], despite it being one of the most common symptoms across neurologic disorders. There is no single, valid and reliable instrument for the assessment or measurement of fatigue. Many scales have been developed, and these scales assess fatigue from a number of perspectives such as its severity, or its impact on lifestyle and associated emotional effects. Most measures of fatigue are self-report measures. The Barrow Neurologic Institute (BNI) Fatigue Scale and the Cause of Fatigue Questionnaire [21] were designed specifically for patients who have experienced a TBI. Other measures were designed for individuals with other types of brain injury or disease, but have now been validated in studies of people with TBI. These include the Visual Analogue Scale for Fatigue (VAS-F) [22], which subjectively quantifies fatigue and energy levels on a likert scale at one point in time; the Fatigue Severity Scale (FSS) [23], a 9-item general fatigue scale used to assess the consequences of fatigue and its impact on daily functioning using a 7-point scale; the Global Fatigue Index (derived from 15 of 16 items of the Multidimensional Assessment of Fatigue) [24]; the Fatigue Impact Scale [25], a 40-item test with three subscales that describe how fatigue impacts cognitive, physical and psychosocial functioning on a 5-point scale; and the Mental Fatigue Scale (MFS) [26], a 15-item test assessing affective, cognitive, sensory and sleep-related symptoms. Scales which have been developed and used to characterize individuals’ subjective sense of fatigue and sleep dysfunction in multiple populations include the Functional Outcomes of Sleep Questionnaire [27], the Pittsburgh Sleep Quality Index (PSQI [28]) and the Epworth Sleepiness Scale [29]. Questions about fatigue/sleep are often included in other self-report assessment instruments as well (e.g., the Beck Depression Inventory, the Profile of Mood States).

While this proliferation of measures reflects a recognition of fatigue as a major clinical problem in many conditions, the information derived from these self-report scales depends on the information collected, and is influenced by the scale developer's conceptualization of fatigue, the targeted clinical population and the responder's interpretation of the questions asked [30]. Therefore, choice of scale can be driven by a number of factors, including what aspect of fatigue is of interest, reliability and validity, and target population. Severity, frequency, impact, phenomenology and functional outcome are variably assessed across fatigue instruments. Thus, choice of the most appropriate measure is not straightforward, and lack of consistency of definition across measures further complicates investigation and understanding of fatigue. For example, some measures instruct respondents to rate their fatigue over a period of time, such as over the last three weeks (e.g., the FSS). Thus, these measures can be thought of as assessing ‘trait’ fatigue, or the extent to which an individual is disposed to fatigue [31]. Other measures assess ‘state’ fatigue, or fatigue in-the-moment (e.g., the Visual Analogue Scale for Fatigue). While one might hope that trait fatigue measures reflect some sort of average or integration of an individual's state fatigue ratings over time, this is unlikely to be the case. Rather, it is more likely that when an individual is asked to rate his/her fatigue over a period of time such as the past 3 weeks, other factors that may or may not be related to fatigue are incorporated into the individual's rating (e.g., sleep quality, depression and social interaction) [32]. Thus, while state and trait measures of fatigue have the potential to provide different types of information about fatigue, state measures may be less biased by other factors than trait measures. More generally, it is important to bear in mind the differences between state and trait measures of fatigue when interpreting the results of these tests.

## Fatigue as symptom of TBI

Fatigue has been defined as ‘a subjective lack of physical and/or mental energy that is perceived by the individual or caregiver to interfere with usual and desired activities’ [33]. While this definition was created to describe fatigue in multiple sclerosis, it captures several salient aspects of fatigue in clinical populations such as mTBI: fatigue is strongly subjective, it has both a physical and a mental component, and it interferes with an individual's life. Indeed, research shows that fatigue is common in numerous clinical populations, that it significantly affects quality of life [34,35] and that it is often given as the primary complaint in physician visits [36].

In fact, fatigue has been acknowledged to be a multidimensional concept by the World Health Organization's International Classification of Functioning, Disability and Health (1976). This is because fatigue interacts with brain injury or disease to affect body function and structure, as well as the activity and participation of the patient. Many subtypes of fatigue have been identified, including cognitive fatigue, physical fatigue, emotional fatigue and stress fatigue (Table 1). This paper will focus primarily on cognitive fatigue, although all aspects of fatigue have an effect on activity and participation and are in most diseases related to health status and disease severity. In addition, psychosocial factors have an influence on fatigue and on activity and participation.

**Table 1.** **Definitions of various dimensions of fatigue.**

Fatigue	A subjective lack of physical and/or mental energy that is perceived by the individual or caregiver to interfere with usual and desired activities. Perceptions of fatigue refer to subjective sensations of weariness, exhaustion, increasing sense of effort, or a mismatch between effort expended and actual performance

Cognitive fatigue (mental fatigue)	A transient increase in mental exhaustion resulting from prolonged periods of cognitive activity. Cognitive fatigue can be described as feelings of mild to extreme mental exhaustion which can last anywhere from several hours to days and is often felt as a rebound effect after mental exertion

Physical fatigue (physiological fatigue)	The transient inability to maintain optimal physical performance following physical exercise. The contractile properties of muscles are reduced, and continued exertion is difficult until the muscle is allowed to rest. Includes both a central and peripheral component

Central fatigue	A form of fatigue that is associated with changes in the synaptic concentration of neurotransmitters within the CNS, including the brain and spinal cord, which affects exercise performance and muscle function and cannot be explained by factors that affect muscle function. It is linked to neurotransmitter systems in the brain, primarily involving serotonin, noradrenaline and dopamine, which are linked to arousal, sleepiness and mood. When central fatigue develops, the CNS is unable to sufficiently enervate the muscles to maintain optimal muscle activation, resulting in reduced muscle force as well as generalized feelings of tiredness, loss of drive and sleepiness. Central fatigue tends to be of a lower intensity and duration than peripheral fatigue

Peripheral fatigue	A form of fatigue resulting from internal changes in the skeletal muscles and the neuromuscular junction during exercise such that the muscles are unable to continue producing the same level of force. Peripheral fatigue is caused by a decrease in the availability of energy resources within the muscle, and with an increase of lactic acid and other metabolites within the muscle (causing a burning sensation)

Emotional fatigue	A form of fatigue encountered when dealing with life events that cause intense emotional reactions such as problems in a relationship, the illness or death of a loved one or problems with a child. These emotional situations engender feelings of fear, dread, confusion, grief or anger that are perceived as overwhelming and that cause generalized stress to the body. This state of constant tension can drain energy and leave one feeling drained of emotional and physical resources

Stress fatigue	A form of fatigue occurring as a result of chronic stress that can be related to jobs, managing family obligations or dealing with unexpected setbacks. If this stress is not managed, it can result in fatigue, to damage to the heart and blood vessels and a number of other serious diseases. Stress fatigue can be related to emotional fatigue, but it can occur independently. Exercise, meditation, biofeedback training or engaging in absorbing hobbies can all help to relieve stress and increase emotional wellbeing

Fatigability	An objective change in performance. Fatigability is defined as the magnitude or rate of change in a performance criterion relative to a reference value over a given time of task performance or measure of mechanical output

To further focus this review, we will concentrate on primary fatigue rather than fatigue that is secondary to some other factor. Secondary fatigue is fatigue that is due to, for example, poor sleep quality or chronic inflammation. In contrast, primary fatigue is due to the brain injury itself, and is not due to some intermediate factor. For instance, fatigue has been found to contribute uniquely to disability status and headache severity among adults with chronic TBI in the community, independent of injury severity, executive dysfunction and depression [37,38]. Because of its unique contribution to disability status, this is an example of primary fatigue. Another example is from a study following individuals with mTBI over 10 years: the most common long-term complaints were fatigue, insomnia and exhaustion [39], suggesting that fatigue is one of the primary sequelae of mTBI. Although it is a significant and prevalent problem, however, the etiology of fatigue is poorly understood (for review see [40]).

## Cognitive fatigue versus physical fatigue

One attempt to grapple with this amorphous construct has been to classify fatigue as either physical (physiological) or cognitive (mental). In this review, we will concentrate on cognitive fatigue. Kluger, Krupp & Enoka [31] propose a unified taxonomy and a novel assessment approach to address distinct aspects of fatigue. In their model, they use the term ‘fatigue’ to refer to subjective sensations, and ‘fatigability’ or ‘performance fatigability’ to refer to objective changes in performance. Perceptions of fatigue refer to subjective sensations of weariness, exhaustion, increasing effort, or a mismatch between effort expended and actual performance. In contrast, fatigability is defined as the magnitude or rate of change in a performance criterion relative to a reference value over a given time of task performance or measure of mechanical output. Of note, perceptions of fatigue and fatigability are not only distinct but also potentially independent. In Parkinson's Disease, for example, Lou *et al*. [41] found evidence of objective decrements in motor performance that did not significantly correlate with perceived fatigue. We will return to this at the end of this section.

Although fatigue has a strong subjective element, subjective, self-report measures are notoriously prone to bias. There have, therefore, been several attempts to examine cognitive fatigue objectively, generally by inducing cognitive fatigue and then examining behavioral performance (see [20] for review). Two major approaches for inducing cognitive fatigue have been developed: cognitive fatigue induced over a prolonged period of time (e.g., over the course of a work day or neuropsychological battery) and cognitive fatigue induced through sustained mental effort (e.g., examining behavioral performance during the course of a neuropsychological task requiring constant cognitive effort) [20].

In the TBI literature, the *sustained mental effort* approach has been used almost exclusively (with mixed results), and for that reason, we focus on it here. For example, Ziino and Ponsford [42,43] published two studies in which they investigated the effects of performing a 45 min. long vigilance task on several indices of mental fatigue. In addition to self-report measures, such as the FSS, they also asked subjects to perform a complex selective attention task (C-SAT) before and after the vigilance task, with the idea that performance on the C-SAT might represent a more objective measure of mental fatigue. Although correlations were found between the FSS and performance on the second administration of the C-SAT (i.e., after the vigilance task) [42], no such correlations were evident between subjective reports of fatigue (e.g., the FSS) and *change* in performance on the C-SAT from before to after performance of the vigilance task [43]. This suggests that fatigue may be associated with selective attention, but not with changes in selective attention following sustained performance on a cognitively demanding task. This failure to find a consistent relationship between subjective reports of fatigue and objective, behavioral metrics is all too common in the literature on fatigue. However, there are some exceptions which have led to the proposal of three cognitive fatigability domains: attention fatigability (using tests such as the Ruff 2 & 7 Selective Attention Test), executive fatigability (using tests such as the Stroop Color Word Test) and psychomotor fatigability (using tests such as the Digit Symbol Substitution test from the WAIS [44]). For example, Belmont, Agar and Azouvi [7] reported that participants with TBI reported a higher baseline level of fatigue than healthy controls (HCs), and also performed significantly more poorly on a test of selective attention. This lends support to the idea of attentional fatigability in TBI. Similarly, performance on the auditory Psychomotor Vigilance Task was reported to be impaired in patients with TBI relative to HCs, with evidence of slower response times, increased response variability and more attentional lapses [45]. Self-reported fatigue was associated with a more global impact on attention performance on this test. This supports the idea of both attentional fatigability and psychomotor fatigability in TBI. A study by [44] supported the idea of psychomotor and executive fatigability with the finding that patients with mTBI reported significantly more mental fatigue and performed worse on psychomotor and executive tests than HCs. Of note, they found that the cognitive fatigability measures were not influenced by depression or sleep disturbances, although these factors did influence self-reports of fatigue. Other studies found that subjective mental fatigue correlated primarily with objectively measured information processing speed [46,47]. Thus, there are some cases in which subjective reports of mental fatigue have been found to correlate with objective behavioral measures. While several of these involve correlations between cognitive fatigue and behavioral tests that load on attention, correlations have also been found between cognitive fatigue and the domains of executive function and psychomotor function, as well as processing speed.

Correlations between cognitive fatigue and behavioral performance are encouraging because we intuitively expect to find them. When we are cognitively fatigued, we feel more sluggish and less sharp. It therefore follows that cognitively fatigued individuals should take more time to respond and make more errors. When such correlations are reported, we are reassured and our notions of fatigue are supported; when such correlations are not found – and the literature on fatigue is rife with such null effects dating back to 1904 [48] – it points out the fact that our models of fatigue are quite primitive. In the next section, we review models of fatigue in mTBI.

## Models of fatigue in mTBI

Research into fatigue in mTBI has attributed fatigue to several factors. These fall into several categories, and while there are currently few formal models of fatigue, these categories serve as a starting point for the creation of models of fatigue. In this section, we have grouped the research we reviewed into studies that have proposed that fatigue is the result of increased effort, that fatigue is an indication of neuro-inflammation, that fatigue is due to psychological risk factors and that fatigue is a function of sleep disturbances.

### Fatigue as an indication of effort

The idea that fatigue is an indication of increased effort following mTBI derives from the idea that a TBI, even a mild one, causes structural damage to the brain that disrupts the cognitive system. This disruption results in inefficient information processing, which means that the brain must expend more energy (effort) in order to perform a given amount of cognitive work. In its simplest form, this line of thinking then proposes that, because the brain is constantly working harder, it is prone to fatigue (in much the same way that an overworked muscle becomes fatigued). This was, for example, the view of Van Zomeran *et al*. [49], who suggested that TBI leads to cognitive impairment, and that individuals who have sustained a TBI are therefore required to use more cognitive resources, or mental effort, to perform cognitive tasks. This increased mental effort resulted in cognitive fatigue. Similarly, some authors [7,42,49] have proposed the ‘coping hypothesis’, which proposes that fatigue is related to the mental effort necessary to overcome attention difficulties and slowed processing. One line of support for this view derives from their studies, which have found significant correlations between mental effort, attentional performance and subjective fatigue in TBI groups (see also the section above reviewing executive, attentional and psychomotor fatigability). Another line of support comes from studies that have investigated brain structure following mTBI. For example, Wäljas *et al*. [50] found that their sample of individuals with mTBI reported more fatigue than HCs, and that the individuals with mTBI also had decreased fractional anisotropy, which is a measure of white matter integrity (using diffusion tensor imaging). Thus, these results support the contention that mTBI results in measurable, persistent damage to the white matter in the brain.

The idea that the brain must work harder following mTBI is supported by several lines of evidence. For example, studies measuring cortical activation using electroencephalography (EEG) have shown that fatigue (in HCs) is associated with changes in θ-band activity, and also with changes in the location of α power [51]. These results show that there are measurable changes in brain activation – results that are consistent with a growing body of literature using fMRI to investigate changes in brain activation after mTBI.

These fMRI studies have shown that persistent post-concussive symptoms are associated with increased activation in attentional areas such as the anterior cingulate cortex (ACC) and with decreased activation in the ‘default network’ (DN), an interconnected group of brain structures that are hypothesized to be part of a functional system [52]. Increased activation in the ACC suggests that either individuals with mTBI are using more attentional resources, or that the ACC must work harder to support task-related processes in individuals with mTBI than in HCs. Either of these alternatives would be expected to result in increased mental fatigue. The decreased activation of the DN is consistent with this interpretation. Studies have shown that the brain areas comprising DN are more active at rest than during task performance [53]. Indeed, activation in the DN has been shown to be inversely related to task difficulty such that as a task is made progressively more difficult, there is progressively less activation in the DN [54]. This means that DN activation can be used as a marker for task difficulty – the greater the DN activation, the easier the task – which is particularly useful when investigating differences between clinical populations (such as mTBI) and HCs. Thus, studies showing less DN activation in individuals with mTBI than HCs suggest that the brains of individuals who have sustained a mTBI must work harder than HCs [52,55]. Similar results were shown by Shumskaya *et al*. [56], who found increased functional connectivity in another brain area associated with attention: the dorsolateral PFC. They interpreted this as showing that the brain had to work harder following mTBI, resulting in fatigue. They also found decreased functional connectivity in the motor network (including striatum), which they suggested underlies motor slowing in mTBI.

A variant of this idea was proposed by McAllister *et al*. [57], who found more brain activation in individuals with mTBI, relative to HCs, on a difficult working memory task. This, they proposed, may be due to ‘the ability to activate, modulate or allocate processing resources in response to gradations of processing load [that] may be impaired in the post-acute period after mTBI’. That is, that mTBI leads to difficulty in regulating brain activation rather than simply to inefficient processing. However, while the mechanism may be different, the end result of an inability to appropriately modulate processing resources would be an increase in effort because individuals with mTBI would be unable to use fewer processing resources when fewer were required. Thus, based on the logic above, this model is also consistent with the idea that fatigue in mTBI is due to increases in the effort expended to process information.

A more recent variant of this idea is was proposed by Dobryakova *et al*. [58], who suggested that fatigue was the result of an imbalance between effort and reward [58]. As with other researchers, Dobryakova *et al*. proposed that TBI results in structural damage and that the brain must work harder in order to overcome inefficiencies in information processing. Dobryakova *et al*. went on to observe that the striatum of the basal ganglia is implicated in fatigue following brain damage (as well as brain disease) [59–63]. For example, in a study that investigated the neural substrates of fatigue in moderate to severe TBI, Kohl *et al*. [61] demonstrated that activation in the caudate of the basal ganglia showed an abnormal increase across successive blocks of an attentionally demanding task, consistent with the idea that the caudate is sensitive to cognitive fatigue. A separate line of research has shown that the caudate is also important in processing motivation and reward [64,65]. Thus, the caudate nucleus appears to be important for the processing of motivation, reward and cognitive fatigue. Dobryakova *et al*. [58] therefore hypothesized that because the brain has to expend more effort to perform cognitive tasks following an injury (e.g., mTBI), the normal balance between effort and reward has been disrupted, and the striatum (specifically the caudate) plays a critical role in detecting this disruption. Furthermore, this disruption between effort and reward detected by the caudate may be experienced by the individual as ‘cognitive fatigue’. This idea is further supported by results showing that the thalamus and the medial prefrontal cortex (PFC) – both of which are part of the reward circuitry in the brain that includes the striatum – are associated with fatigue in mTBI [66].

Taken together, the studies reviewed above show that mTBI can result in measurable, persistent damage to the brain, that the brain has to ‘work harder’ (as shown by increased activation) following a mTBI and that fatigue is associated with areas in the brain implicated in the processing of reward and motivation.

### Fatigue as an indication of neuro-inflammation

Several studies have investigated the link between neruo-inflammation and fatigue. This is because it is now known that the CNS generates inflammatory mediators in response to injury. These mediators include pro-inflammatory cytokines, prostaglandins and free radicals, which in turn induce chemokines and adhesion molecules, recruit immune cells and activate glial cells (for a review see [67]). Indeed, following a TBI, it has been shown that there is low-grade neuro-inflammation with downregulation of astrocyte glutamate transporters and Na^+^/K^+^ ATPase activity [68]. Pro-inflammatory cytokines and the metabolic changes that they produce can cause fatigue [69]. In severe TBI, serum cytokine levels have been shown to be elevated in the months following the injury and this has been associated with worse functional outcomes [70]. These data suggest that pro-inflammatory cytokines may have an effect on fatigue levels in TBI, particularly in more severe cases. These effects are likely to be relegated to the acute and sub-acute phases of TBI, since it is during this time that the initial trauma and secondary inflammation cascades lead to increases in cytokine levels [69].

However, while fatigue may well be associated with neuro-inflammation, particularly after severe trauma, pro-inflammatory cytokines are unlikely to underlie chronic fatigue following mTBI. This is because one would expect inflammation to be proximal to the injury (rather than chronic) [69], and also because there is little evidence supporting the link between fatigue and neuro-inflammation in mTBI. Indeed, Su *et al*. found that fatigue does not appear to be due to neuro-inflammation as measured by C-reactive protein levels in mTBI [71].

There is a clear need for increased research in the area of neuro-inflammation in mTBI. Based on the current literature, it is difficult to determine the extent to which fatigue in mTBI is related to neuro-inflammation. It may be that following even a mTBI, levels of pro-inflammatory cytokines are chronically elevated, leading to chronic fatigue. Alternatively, it may be that fatigue associated with neuro-inflammation is confined to the acute and subacute period of recovery (because neuro-inflammation gradually abates after the trauma), and that chronic fatigue in mTBI is due to a different cause. Further work is required to adjudicate between these possibilities.

### Fatigue as an indication of psychological processes

Fatigue has also been conceived of as a psychological process that protects the cognitive system from exhaustion. Thus, central physical fatigue has been proposed to be a mechanism designed to prevent the body from over exertion, which might eventually lead to upsetting homeostasis and causing irreparable damage to muscles and organs [72]. In a similar vein, mental fatigue may protect individuals from frustration and failure by causing them to cease performing mental tasks before frustration and failure become severe [73]. This view has strong parallels with the view that fatigue is an indication of effort (above) in as much as both propose that fatigue is a sort of signal that the brain generates to tell itself to stop. Moreover, the view that fatigue is protective also seems to entail the idea that the cognitive system must work harder following mTBI. The main difference between the idea that fatigue is protective and that fatigue is an indication of effort therefore appears to be one of emphasis, with the former emphasizing the avoidance of negative outcomes (muscle damage or frustration).

A rather different conception of fatigue is that it is related to modifiable psychological risk factors. For example, individuals who have sustained mTBI but who are psychologically resilient report less fatigue than individuals who have mTBI but who are less resilient [74]. Similarly, Losoi *et al*. [75] found that while individuals who had sustained a mTBI initially reported more fatigue than HCs, this fatigue resolved after 1 month except in those individuals who had modifiable psychological risk factors such as traumatic stress, depression and/or low resilience. For these individuals (with modifiable psychological risk factors), fatigue persisted for up to a year after the mTBI. This is certainly an interesting line of work, and it highlights an important aspect of fatigue research. In both these studies, the instrument used to assess fatigue was the BNI [21], which is a 10-question survey that asks the respondent to rate fatigue-related aspects of their mental life since their brain trauma. However, as is common in fatigue assessment instruments, not all of the questions assess fatigue: in the case of the BNI, several questions assess aspects of sleep, attention and alertness. While questions relating to sleep, attention and alertness do allow the BNI to capture the wide-ranging impact fatigue has on an individual's life, the inclusion of these questions also makes it difficult to interpret the resulting score. Some of the resulting ‘fatigue score’ will be related to sleep quality, some will be related to attentional processes (which touch nearly every aspect of cognition), and some will be related to energetic (alertness) levels. Moreover, the BNI asks respondents to rate these abilities over the period of time since their trauma, thus requiring them to integrate their judgment of their mental abilities over an ever-increasing time interval. Therefore, if an individual felt s/he had trouble concentrating since the trauma (an aspect of attention), or had had less energy since the trauma, this would be reflected in the BNI score.

This conflation of ideas – mental fatigue, sleep, attention and alertness – is not specific to the BNI. On the contrary, it is found (to a greater or lesser extent) in virtually all the instruments that have been developed to assess fatigue. And this conflation of ideas is one very plausible reason why fatigue scores are so often correlated with other measures, such as depression or, in the research cited above, resilience. Indeed, the resilience questionnaire used (The Resilience Scale [76]) included questions assessing concentration and energy levels – which may underlie some of the shared variance between the BNI scores and the resilience scores. This underscores the importance of carefully defining what one means by ‘fatigue’ both during assessment and during the interpretation of one's findings.

### Fatigue as an indication of sleep problems

It is important to distinguish between actual sleep disorders that can occur after TBI, and the subjective experience of fatigue. Sleep disturbances are frequently identified following TBI, affecting 30–70% of persons, and often occur after mTBI [77]. Insomnia (i.e., difficulties falling or staying asleep), fatigue and sleepiness (increased sleep need) are the most frequent sleep complaints after TBI. *Sleepiness* is generally caused by not enough proper, restful sleep or a lack of stimulation, and results in the need for increased sleep. *Fatigue* is usually a more chronic condition than sleepiness. People who suffer from fatigue feel they lack motivation and energy. Even though fatigue and drowsiness are not the same, drowsiness or the desire to sleep, is a common symptom that accompanies fatigue. Apathy may also accompany fatigue. However, sleep disorders such as sleep apnea (obstructive and central), narcolepsy (with or without cataplexy), periodic limb movement disorder, post-traumatic hypersomnia and parasomnias may also occur after a TBI. Potential causes include injury to brain regions associated with sleep-wake regulation and/or disruption of circadian timing of sleep [78]. Secondary factors, such as depression, anxiety and pain, may also impact on sleep.

Diagnosis of sleep disorders after TBI is made using polysomnography, multiple sleep latency testing or actigraphy. Treatment is based on the specific disorder, and may include the use of medications, continuous positive airway pressure, or behavioral modifications. It has been reported, however, that treatment of sleep disorders in individuals with TBI often does not improve sleepiness or cognitive functioning [79]. Individuals with TBI have been shown to report significantly poorer sleep quality and higher levels of daytime sleepiness relative to noninjured controls; in addition, sleep diaries have revealed longer sleep onset latency, poorer sleep efficiency, longer sleep duration, more frequent daytime napping, earlier bedtimes and greater total sleep duration [80].

Importantly, while insomnia and fatigue often co-occur following a TBI (9–22%), and are both associated with poorer quality of life, they are affected independently by a variety of factors, especially psychopathology and sleep quality [81]. While insomnia without fatigue is relatively rare (2–3%), post-TBI fatigue without insomnia occurred in 21–23% of individuals assessed. In one study by Beaulieu-Bonneau and Morin [82], fatigue was noted to be a more prominent symptom than sleepiness when assessed 1–11 years post TBI. Secondary factors were associated with poorer sleep quality, while greater injury severity was associated with a need for longer sleep time. In fact, in one model of fatigue following TBI, Ponsford, Schonberger & Rajaratnam [83] propose that fatigue after TBI is a *cause*, not a *consequence*, of anxiety, depression and daytime sleepiness, which in turn may exacerbate fatigue by affecting cognitive functioning. This model proposes that to alleviate fatigue, each factor must be addressed individually.

Very little of the research that has been conducted on the relationship between sleep and cognitive fatigue in TBI has specifically investigated mTBI. One exception is Schiehser *et al*. [84] who investigated this relationship in veterans with mild–moderate TBI. They found that poor sleep quality (as assessed by the PSQI) predicted cognitive fatigue (as assessed by the cognitive subscale of the MFIS). Thus, there is some evidence for a relationship between poor sleep quality and cognitive fatigue in mild–moderate TBI, though more work is required to establish the nature of this relationship. While Schiehser *et al*. have shown that the MFIS and the PSQI share some common variance, it may be that both poor sleep and cognitive fatigue proceed from the same cause, or it may be that one causes the other. In other TBI populations, it appears that poor sleep quality or insomnia is not the cause of fatigue. The use of targeted statistical approaches (e.g., mediation analyses) and careful use of neuropsychological instruments (that do not contain items that assess overlapping constructs) are required to establish whether this is also the case in mTBI.

### Summary of models

In the above, we have reviewed four ways that researchers have attempted to understand cognitive fatigue in mTBI. This is not an exhaustive list of models of fatigue, but rather this is one way to organize the research reviewed here. Broadly, this research can be understood as linking fatigue to an increase in the effort necessary to perform cognitive work, linking fatigue to neuro-inflammation, linking fatigue to psychological processes or linking fatigue to sleep quality. Of these models, there appears to be little support for the idea that chronic fatigue in mTBI is due to neuro-inflammation, though it is entirely possible that fatigue experienced during the acute and subacute periods may be at least partially due to this cause. However, this conclusion may change as more research is undertaken to better understand the relationship between neuro-inflammation and fatigue in mTBI. The idea that fatigue in mTBI is linked to psychological processes such as resilience also represents an area where more research could produce illuminating results. However, until more research is conducted, it is not possible to rule out the possibility that the linkage between psychological processes and fatigue is due to the fact that the instruments used to assess fatigue and psychological processes such as resilience both contain items that assess common constructs.

Although fatigue and sleep problems appear to be relatively independent in individuals with more severe TBI, in mTBI it has been shown that sleep quality predicts cognitive fatigue [84]. This is an important result, since individuals with mTBI can be trained to improve their sleep habits, thereby improving their sleep quality (see below). Based on the association reported by Schiehser *et al*. one would predict that improved sleep quality would result in reduced cognitive fatigue, and, to foreshadow a section below, Ouellet and Morin have provided partial support for this by showing that improving sleep habits resulted in less general and physical fatigue [85]. Thus, more work is clearly warranted to better understand the links between sleep quality and fatigue in mTBI.

In the research reviewed here, the model with perhaps the most support was the model linking fatigue to an increase in the effort necessary to perform cognitive work. Unlike some of the other models that rely on correlations of two self-report measures, this model links self-reported fatigue to objectively measured changes in the brain. This is a strong advantage, since there is often overlap in the constructs assessed by self-report instruments that are designed to assess such things as, for example, ‘fatigue’ and ‘resilience’ (see above). As with the other models, more work needs to be done to support this model, yet of the four models reviewed here, it is our view that this model represents the most promising way forward.

## Treatment

There has been relatively little work done to develop interventions for fatigue following TBI in general, and mTBI in particular, and at this time there are no evidence-based treatments with sufficient evidence to be recommended or contraindicated for posttraumatic brain injury fatigue [86,87]. In assessing individuals with mTBI who report fatigue, the clinician should assess all possible contributing factors (e.g., attention/processing speed, medications, pain, mood and sleep disturbance) and provide treatment for these accordingly (see below), as improvement in any of these symptoms may lead to at least some improvement in symptoms of fatigue. Further, it is important to discuss with the individual the need to regulate their lifestyle so as not to exacerbate their cognitive and physical symptoms, including fatigue (i.e., reduce work hours, modify pace or demands of activities, reduce distractions and limit multitasking). Physical conditioning programs can reduce physical fatigue and promote well-being, although they may not improve other types of fatigue. Lastly, sleep hygiene techniques may be helpful in improving sleep and decreasing fatigue in individuals with mTBI [85].

Several treatments have been described to help address posttraumatic fatigue and are reviewed below. These include pharmacological, cognitive and behavioral interventions.

### Medications

As has been described, TBI may result in long-lasting postconcussive symptoms, such as cognitive fatigue and concentration difficulties, and this is often the main impediment in returning to work or school. While there is currently no effective treatment for chronic cognitive fatigue, methylphenidate is the most common medication that has been used to help combat cognitive fatigue and enhance cognitive function in this population. This treatment is based on the notion that TBI results in a need for increased effort on the part of an individual to maintain pre-injury levels of performance and help overcome attentional difficulties and slowed processing speed (see above). Moreover, because methylphenidate is a dopamine agonist, it results in an increase in the levels of dopamine in the brain (particularly in prefrontal areas), suggesting a link between dopamine levels and fatigue in TBI [88]. Johansson *et al*. [89] found that methylphenidate significantly reduced cognitive fatigue, as evaluated by the Mental Fatigue Scale, but did not help with pain in 28 individuals with mTBI. The effects were dose dependent, with normal dose (20 mg tid) more effective than low dose (5 mg tid). These researchers [90] also treated 30 individuals with long-term postconcussion symptoms after mild–moderate TBI who had reported positive effects with methylphenidate in the initial phase of study. They were given methylphenidate for a further 6 months in this follow-up study, and results indicated reduced cognitive fatigue and improved cognitive functions in these individuals.

Other medications may help treat an underlying sleep disorder, and in turn, lead to improvements in cognitive functioning. Modafinil is a wake-promoting drug and an example of a smart drug that has been used like a nootropic (cognitive enhancing drug). It has been used for treating excessive sleepiness associated with narcolepsy, obstructive sleep apnea and shift-work disorder. Perhaps because it is common to conflate sleepiness and fatigue, Modafinil has also been used as a potential treatment for fatigue in individuals with TBI, but without success. One randomized controlled study [91] showed no evidence of reduction in subjective fatigue in a general TBI sample (i.e., a sample not limited to mTBI), although a trend toward reduction in daytime sleepiness was reported (at week 4, but not week 10). A more recent randomized control trial [92] showed reduction in self-reported daytime sleepiness (using the Epworth Sleepiness Scale), but not in fatigue (using the FSS) in a mixed sample of individuals with mild to severe TBI. Similarly, donepezil has not been found to reduce fatigue in individuals with moderate to severe TBI [93]. Furthermore, hypnotic and benzodiazepine-like compounds including zolpidem and zopiclone are contraindicated despite their use in the treatment of chronic, primary insomnia, as they are associated with impaired cognitive function and reduced daytime alertness, as well as hallucinations, sleepwalking and altered sleep architecture [94]. Piracetam is a nootropic medication, and has been shown to be helpful for reducing post-TBI fatigue [86,95].

Melatonin has been shown to improve latency to sleep, sleep efficiency, morning alertness and arousal, and quality of life in chronic patients with insomnia over the age of 55 [96,97]. Only one study has evaluated the efficacy of melatonin in individuals with TBI [98]. While no significant improvements were found in subjective sleep parameters such as latency, duration, quality or daytime alertness in these seven male TBI patients with chronic sleep difficulties at least 6 months post injury, melatonin was found to have a moderate effect on daytime alertness relative to amitriptyline; fatigue was not directly measured. In another study that investigated ramelteon (a melatonin receptor agonist), preliminary data showed that this drug can lead to improvement in total sleep time and in some aspects of executive functioning as measured by neuropsychological testing in a mixed sample of individuals with mild to severe TBI [99]. Subjective questionnaires were also administered to assess mood, daytime sleepiness and fatigue, and administered at weeks 2–5, but no information was provided as to whether there was a change in fatigue or vigor. No differences were seen between groups (treatment first vs placebo first) on mood, daytime sleepiness or fatigue.

To summarize, three broad classes of medications have been used to help reduce fatigue in TBI: those designed to help with attention/concentration, those designed to help with cognition generally and those designed to help with sleep. Of these, only methylphenidate, a dopamine agonist, has been found to affect fatigue. Medications that help with sleep have not been found to reduce fatigue, and nootropic medications (which were designed to help cognition generally) have produced mixed results. This pattern of results underscores other evidence that fatigue in TBI is independent of sleep quality, and suggests instead that fatigue may be related to dopaminergic areas of the PFC.

### Therapeutic Interventions

Several randomized controlled studies have been identified examining nonpharmacological interventions for fatigue management after TBI and other brain injuries (stroke, subarachnoid hemorrhage etc.) [86,100]. These are reviewed below.

#### Cognitive behavioral interventions

There is some evidence to suggest that cognitive behavioral therapy (CBT), alone or in conjunction with other treatments, may be helpful in reducing postinjury fatigue and is worthy of further study [85,87,101–103]. Ponsford *et al*. point out the need to help patients to regulate their lifestyle ‘to live within cognitive and physical limitations’ (p. 231). Strategies include reducing work hours, pacing activities, reducing the demands of activities, taking frequent rest breaks, reducing distraction and avoiding situations that require multitasking. They endorse the use of general cognitive behavior therapy techniques to address psychological issues related to making such changes in lifestyle. Ouellet and Morin indicate that any effective intervention approach should involve multiple components, including biological, psychological and social. They suggest that a combination of gradual physical reconditioning, cognitive restructuring, cognitive retraining, anxiety-reducing techniques and education around proper energy management techniques, with a gradual increase in social or occupational activities, could prove beneficial. Using a single-case experimental design, they tested the efficacy of a cognitive-behavioral therapy for insomnia in individuals with mild to severe TBI in an outpatient rehabilitation center [85]. The program consisted of an 8-week CBT that included stimulus control instruction (to re-associate the bedroom and bedtime stimuli with sleep rather than with frustration and anxiety), cognitive restructuring (related to sleep and insomnia), sleep restriction (limiting time spent in bed to the actual time spent sleeping), sleep hygiene education and fatigue management skills training. Results indicated clinically and statistically significant reductions in total wake time and sleep efficiency in eight of 11 participants, which was maintained at both 1 month and 3 months follow-ups. Sleep improvement was associated with fewer symptoms of general and physical fatigue.

Rees and Bellon treated 20 patients with persistent post concussive symptoms 1 year after mTBI (ages 18–52). No participant had had a prior TBI. Each subject participated in individual client centered counseling and CBT for 2 hours every 2 weeks for 6 months, then for up to 2 hours monthly for 18 months. The intervention included education, client centered therapy and CBT which involved sharing information about post concussion difficulties; using PCS questionnaires to identify PCS; rank ordering symptoms by participants to drive counseling, problem solving and symptom validation; development of compensatory strategies for prioritized symptoms; sharing information about mood states and their impact on behavior; discussion and feedback; developing ability to attend, persist at tasks and set appropriate goals; and development of strategies to improve participation in physical, cognitive and social activities. At baseline, 100% of the sample reported physical fatigue and cognitive fatigue, as well as difficulty making decisions and anxiety. At 2 years follow-up, there was a significant reduction in physical fatigue (endorsed by 40% of the sample) although cognitive fatigue remained a problem (endorsed by 90% of the sample). Difficulty making decisions and anxiety also remained problematic. On the Beck Depression Inventory, significant reductions were noted on a number of items, including loss of energy, changes in sleeping pattern and tiredness/fatigue. This study suggested that CBT in combination with client-centered therapy was an effective intervention for individuals with PCS associated with mTBI, although some symptoms, including cognitive fatigue, irritability, poor frustration tolerance and feeling overwhelmed were not affected. More work was recommended given the lack of a control group in this study.

Hodgson *et al*. provided a cognitive behavioral intervention specifically designed for managing social anxiety following acquired brain injury at least 12 months prior to beginning therapy (no further details provided) in 12 individuals with evidence of social anxiety or social phobia. These individuals were allocated to a treatment group or a waitlist group, using a case–control procedure. The core CBT methods in the treatment program included relaxation, cognitive strategies, graded exposure and assertiveness skills training. Participants were seen for one hour per week for 9–14 weeks. Therapy was modified (to address TBI-related symptoms of impaired attention, concentration and fatigue) by having shorter sessions with more frequent breaks, frequent repetition of information and use of visual aids, session summaries and audio tapes; simplified cognitive strategies were also used. Outcome measures included The Social Phobia and Anxiety Inventory [104], the Hospital Anxiety and Depression Scale [105], a self-esteem inventory [106] and the Profile of Mood States [107). Results indicated significant improvements in general anxiety, depression and a transient mood measure (tension-anxiety) at post-treatment and at 1 month follow-up. Of note, fatigue-inertia (a measure on the Profile of Mood States) also improved in the treatment group although there was not a statistically significant difference for those receiving therapy in comparison to those in the waitlist group.

Various measures of fatigue have been reported to improve after a course of CBT, although they vary among studies. By the very nature of this type of therapy, psychological processes that might be affecting a person's behavior are explored; in this case, the role of fatigue as a signal either indicating too much effort is leading to frustration or as a preemptive protective factor can be explored. In addition, the role of resiliency and other modifiable psychological risk factors are considered.

#### Mindfulness-based stress reduction

Mindfulness-based interventions are aimed at reducing psychological symptoms of distress and enhancing quality of life, and have been applied in various settings in both mental healthcare and somatic healthcare. Mindfulness-based stress reduction (MBSR) is an educational program designed to improve attention and cognitive flexibility, increase brain neural connectivity, and help individuals to better cope with their difficulties [100]. It has been used to treat patients with a wide range of conditions, such as stress, depression, pain and fatigue [108]. The goal of the intervention is to cultivate an open-minded and nonjudgmental awareness of what is happening at each successive moment of perception, with a focus on internal psychological states and processes (thoughts, feelings, images etc.), proprioceptive information from the body and external stimulation of the senses. Studies examining the effects of mindfulness-based stress reduction interventions often report beneficial effects in diverse samples such as medical and premedical students, healthcare professionals, patients with chronic pain, patients with fibromyalgia and cancer outpatients. Improvements have been reported in symptoms of general distress, worry/rumination/anxiety, depressive symptoms, pain and sleep quality [87,109].

Returning to cognitive fatigue in TBI, Johansson, Bjuhr & Rönnback [108] used an educational MBSR program, as the effect of MBSR on cognitive fatigue after TBI (n = 11) or stroke (n = 18) had not previously been studied. The therapy included gentle Hatha yoga (with an emphasis on mindful awareness of the body), a body scan (designed to systematically cultivate awareness of the body, region-by-region, without progressive muscle relaxation) and sitting meditation (awareness of breathing and systematically widening the field of awareness to include all four foundations of mindfulness: awareness of the body, feeling tone, mental states and mental contents). The program consisted of 8 weekly 2.5 hour long group sessions, one day long silent retreat between session six and seven and regular home practice (45 min, 6 days a week). Fifteen individuals participated in an MBSR program for 8 weeks, while the other 14 individuals served as controls and received no active treatment, although they were offered MBSR treatment during the following 8 weeks. Johansson *et al*. hypothesized that, compared with a waitlist group, patients randomly assigned to the MBSR program would experience improvement at 8 weeks in their assessment of cognitive fatigue, measured using the MFS and neuropsychological measures of processing speed, attention and working memory. Their results supported this hypothesis, with reductions in self-reported cognitive fatigue and improvements in psychomotor functioning (Digit Symbol-Coding, Trail Making Test) relative to waitlist controls (who received no active treatment). These results are encouraging, and suggest that MBSR may represent an effective means to reduce fatigue after mTBI. However, because the MFS includes questions that assess attention/concentration, cognitive functions such as memory and processing speed, sleep and emotional control, it is not clear to what extent MBSR is able to specifically reduce mental fatigue.

#### Education/symptom management strategies

Theadom *et al*. [110] note four themes identified by TBI survivors regarding fatigue and sleep difficulties:  making sense of fatigue and sleep after TBI; accepting the need for rest; learning how to rest; and the fact that the need for rest impacts one's ability to engage in life. They indicated that targeted support to understand and manage sleep and fatigue difficulties may be crucial to improving recovery and facilitating engagement in everyday life. Education in these areas should be timely, and revised for relevance over the course of recovery. General stress management has also been identified as a way to reduce fatigue burden and increase quality of life [111]. Communication interventions focused on fatigue management have also been identified by speech-language pathologists, who identified a different set of four fatigue-related themes [112]: intervention structure; client and family strategies; monitoring by both the client and therapist; and lifestyle and daily activities. These strategies are global enough to address multiple models of fatigue, including the impact of increased effort, indications of psychological processes and sleep problems.

#### Working memory training

Björkdahl and colleagues [113] evaluated whether computerized working-memory training after TBI had a significant effect on daily life functions. Both the intervention group and control group underwent standard rehabilitation for 5 weeks. In addition, the intervention group also received working-memory training. The Fatigue Impact Scale score in the intervention group improved significantly after working-memory training compared with pretraining, with no improvement seen in the scores of the control group. In relation to the models (above), improving working memory function might be expected to decrease the effort required to process information and thereby reduce fatigue.  This is for two reasons:  1) working memory processes are central to many aspects of cognition, and also 2) individuals with TBI often experience deficits in working memory.

### Other nonpharmacological interventions

#### Light therapy

Use of bright blue light may reduce post-TBI fatigue because light exerts nonvisual effects, including acute alerting effects, on many biological functions. Use of light therapy has been associated with reduced sleepiness, increased vigilance performance and improved mood in both healthy and patient populations [87]. Blue light (short wavelength) is most effective, presumably due to the key role of photopigment melanopsin (which is sensitive to blue light) in circadian photoreception. Sinclair, Ponsford, Taffe, Lockley and Rajaratnam [114] completed a randomized, placebo-controlled 4-week study of a 45-min home-based treatment with short wavelength (blue) light therapy compared with yellow-light therapy. Assessment of fatigue and secondary outcomes (self-reported daytime sleepiness, depression, sleep quality and sustained attention) were conducted over 10 weeks at baseline, halfway through therapy, at end of therapy, and 4 weeks post treatment. Treatment with high-intensity blue light therapy resulted in reduced fatigue (type unspecified) and daytime sleepiness during the treatment phase, with evidence of a trend toward baseline levels 4 weeks post treatment. These improvements were not noted for self-reported depression or performance on a task of psychomotor vigilance. This study offers some support for a noninvasive, nonpharmacological intervention for post-traumatic fatigue, particularly if this difficulty is related to sleep problems.

#### Interventions involving physical activity

The use of interventions aimed at increasing physical activity has not generally been found to be helpful in improving subjective ratings of fatigue [115–118]. However, this may be due to poor study design and/or poorly defined fatigue outcome measures. For example, the Six-Minute Walk Test was found to be a useful instrument in segregating physical fatigue from cognitive and psychological aspects of fatigue [119], but this test has not been consistently used to asses fatigue in studies of physical activity. More recently, vigorous aerobic exercise training has been shown to reduce physical fatigue in some individuals with TBI [120]. Thus, the results of studies investigating exercise as an intervention to reduce fatigue are mixed. However, even in clinical populations such as multiple sclerosis where exercise has been shown to reduce fatigue [121], the mechanisms underlying this reduction are unclear. Reductions in fatigue could result from better overall fitness, from increased cerebral blood flow and perfusion, or from improvements in cognition and sleep that follow from exercise.

#### Brain biofeedback/stimulation

A few studies have examined the effectiveness of EEG biofeedback [122] and transcranial electrotherapy stimulation [123] on a range of symptoms, including fatigue. Meaningful improvement in occupational and social functioning was endorsed by most participants in the EEG-based therapy relative to a waitlist control. However, a review by Cantor *et al*. [86] determined that the evidence is weak that biofeedback and cranial electrotherapy stimulation is effective in reducing post-TBI fatigue, and also that there is an elevated risk of bias.

More generally, Cantor *et al*. [86] concluded that there is insufficient evidence to recommend any treatment for post-TBI fatigue at this time, based on the existing literature. This was due, in part, to the fact that few interventions have been tested in more than one study, the risk of bias was high in many studies, and because of the large disparity between the scale of the problem and the extent of scientific knowledge about the efficacy of treatment options. As already discussed, there is a lack of precision in our definition and measurement of fatigue and in differing aspects of fatigue. Further, the root cause and mechanisms involved in the presentation of this prominent symptom are not fully understood, as indicated by the number of models that have been proposed to account for post-traumatic fatigue. Some models suggest that fatigue may be seen as primary, perhaps as a result of altered function in neural networks, or secondary to co-occurring conditions such as depression, stress, neuro-inflammation or sleep disturbance, or as a result of compensatory strategies requiring increased mental effort to successfully engage in cognitive activities. It is likely that in some cases, both primary and secondary factors contribute to the presentation of fatigue. Ultimately, the development of effective treatments for posttraumatic fatigue will depend on more accurate measures of fatigue symptoms, further investigation and characterization of the causes and types of fatigue experienced by individuals after mTBI, the identification of covariates that contribute to or explain treatment response and a better understanding of the various mechanisms and models that account for the self-reported fatigue experienced after mTBI. While these factors can be enumerated separately, they are inter-related and interdependent. For example, having a better model of fatigue after mTBI will inform the choice of assessment tool(s) used to measure fatigue; the choice of assessment tool(s) will affect the data obtained; the quality and nature of the data will affect which factors covary with it; and, finally, the quality and nature of the data will also determine whether a given model of fatigue has explanatory power.

### Treatment of modifiable related & risk factors to help improve postconcussive symptoms

There is also evidence to suggest that by treating specific sleep disorders, individuals with TBI show significant reductions in psychiatric symptoms of PTSD and depression, as well as on ratings of quality of life [124], suggesting the importance of addressing this issue. Therefore, when sleep disorders such as sleep apnea, narcolepsy, periodic limb movement disorder and parasomnias are identified, appropriate sleep interventions including the use of medications, positive airway pressure and/or behavioral modifications can be used. It is important to note, however, that the treatment of sleep disorders associated with TBI may not improve subjective impressions of sleepiness [77] or of fatigue.

Counseling/education and early interventions can also be helpful in improving recovery in individuals who report moderate to severe sleep disorders following mTBI in combination with moderate to severe headache, dizziness and psychiatric symptoms [125]. Another recent study [75] examined 74 previously healthy adults who sustained mTBI at 1, 6 and 12 months after injury. Outcome measures included cognition, postconcussion symptoms, depression, traumatic stress, quality of life, satisfaction with life, resilience and return to work. While almost all patients with mTBI (96%) returned to work/normal activities within 1 year, a large percentage of the subgroup who reported mild postconcussion-like symptoms at 1 year had an identified modifiable psychological risk factor at 1 month (i.e., depression, traumatic stress and/or low resilience), and greater post-concussion symptoms, fatigue, insomnia, traumatic stress and depression at 6 months. This illustrates the importance of providing evidence-supported treatment and rehabilitation services for these modifiable risk factors early in the recovery period, even when the etiology of the fatigue not clearly understood.

### Summary of treatments

Overall, while there are no evidence-based treatments for post-TBI fatigue, the extant literature suggests the following:

- Methylphenidate and bright blue light have shown some promise in alleviating mental fatigue;
- Very limited work suggests that CBT and MBSR may help reduce mental fatigue;
- There is equivocal evidence that cranial electrotherapy stimulation may reduce mental fatigue;
- In general, it is important to provide evidence-based treatment and rehabilitation services early in the recovery period, even when the etiology of the fatigue not clearly understood. These should address other, modifiable risk factors (such as psychological or sleep disorders).

Clearly, far more work needs to be done to better understand the mechanisms underlying these treatments. It will also be important to investigate the long-term effects of these treatments and whether there is sustained improvement once treatment has ended. As this research is done, and the mechanisms are better understood, it is to be hoped that more effective treatments for mental fatigue following mTBI will be developed.

## Future perspective

Over the past decade, there have been important advances in the study of cognitive fatigue generally, and in TBI in particular. The tools of cognitive neuroscience, such as fMRI, have allowed us to assess brain function and dysfunction in ways that were once simply impossible. As we look to the future, we will move beyond studies investigating the neural substrates of cognitive fatigue and bring to bear advances in other domains of clinical science. These include the influence of, for example, genetics, neuroinflammation and the microbiome on behavior and how factors such as these may interact with brain function to produce complex cognitive responses such as fatigue. As our understanding of the complexity of the cognitive system grows, we will require increasingly multidisciplinary and collaborative groups to fully investigate our hypotheses.

It is also to be hoped that as our scientific questions grow to include a broader range of systems, our approach will become more translational. That is, as our understanding of fatigue becomes deeper, we should begin to be able to predict the treatments that will effectively treat fatigue, and also to be informed by the relative effectiveness of different treatments. One example of this is the use of a dopaminergic agonist (methylphenidate) to help treat fatigue. In as much as this is an effective treatment for many people, it supports the idea that the dopaminergic system in the brain is associated with fatigue; however, in as much as it does not help all individuals with cognitive fatigue to the same degree, this suggests that fatigue may also be due to factors beyond the dopaminergic system. This type of cyclical information flow between the lab and the clinic will refine and enhance our ability to treat fatigue in mTBI in the future.

## Conclusion

Fatigue is a common, persistent consequence of TBI of any severity, including mTBI. Despite this, to-date fatigue has been inadequately characterized, assessed and managed. Fatigue clearly detracts from quality of life, interferes with the ability to work, interacts with psychiatric symptoms and exacerbates problems with social functioning. Of the models that have been proposed to account for fatigue, the two with the most support are: that fatigue in mTBI results from the increased effort that the brain must expend in order to process information following injury, or that fatigue in mTBI results from sleep disturbances. These models are, of course, not mutually exclusive. The application of the methods of cognitive neuroscience to the study of fatigue has resulted in important advances in our ability to test hypotheses about the causes and effects of fatigue. It is to be hoped that we are finally beginning to gain a better understanding of the etiology, precipitants and risk factors of fatigue. This is critically important work, in as much as it will enable the development of effective treatments, which will benefit the many thousands of individuals who struggle with fatigue on a daily basis.

**Executive summary**

- Cognitive fatigue is a significant problem for many individuals who have sustained a mild TBI (mTBI).
- There are multiple types of fatigue that individuals with mTBI experience, including cognitive and physical fatigue.
- Here, we group the research on cognitive fatigue in mTBI into four categories, or models of fatigue: 1) fatigue as an indication of effort, 2) fatigue as an indication of neuro-inflammation, 3) fatigue as an indication of psychological processes, 4) fatigue as an indication of sleep problems. Of these models, models 1 and 4 have the most support.
- While there are no evidence-based treatments for fatigue, research has been conducted into treatments in several domains.
  - Medications: the efficacy of three broad classes of medication have been investigated to help reduce fatigue in TBI: those designed to help with attention/concentration, those designed to help with cognition generally, and those designed to help with sleep. Of these, only methylphenidate, a dopamine agonist, has been found to affect fatigue.
  - Therapeutic interventions: the efficacy of cognitive behavioral interventions, mindfulness-based stress reduction, education/symptom management strategies, working memory training are reviewed. Reductions in fatigue have been reported, but these reductions are not always easy to interpret because of the instruments used to assess fatigue.
  - Other nonpharmacological interventions: the efficacy of light therapy, physical exercise and brain biofeedback/stimulation are reviewed. These are promising approaches that may prove useful in alleviating fatigue in the future.
- In general, it is important to provide evidence-based treatment and rehabilitation services early in the recovery period, even when the etiology of the fatigue is not clearly understood. These services should address other, modifiable risk factors (such as psychological or sleep disorders).
- Future research should investigate influence of factors such as genetics, neuroinflammation and the microbiome on behavior, and how factors such as these may interact with brain function to produce complex cognitive responses such as fatigue.
